# Prominent Levels of the Profibrotic Chemokine CCL18 during Peritonitis:* In Vitro* Downregulation by Vitamin D Receptor Agonists

**DOI:** 10.1155/2018/6415892

**Published:** 2018-04-04

**Authors:** Marta Ossorio, Virginia Martínez, Maria-Auxiliadora Bajo, Gloria Del Peso, Maria-José Castro, Sara Romero, Rafael Selgas, Teresa Bellón

**Affiliations:** ^1^Nephrology Service, Hospital La Paz Institute for Health Research (IdiPAZ), La Paz University Hospital and IRSIN, Madrid, Spain; ^2^Hospital La Paz Institute for Health Research (IdiPAZ), Madrid, Spain

## Abstract

Peritoneal dialysis (PD) is used as a renal replacement therapy, which can be limited by peritoneal membrane ultrafiltration failure (UFF) secondary to fibrotic processes. Peritonitis, a frequent complication of PD, is a major risk factor for peritoneal membrane fibrosis and UFF. Low peritoneal levels of the chemokine CCL18 are associated with preservation of peritoneal membrane function in PD. Given that CCL18 is involved in fibrotic processes and recurrent peritonitis, it is a risk factor for peritoneal membrane failure; thus, we evaluated CCL18 concentrations in peritoneal effluents from patients undergoing peritonitis episodes. Pharmacological interventions aimed at diminishing the production of CCL18 were also explored. Fivefold higher CCL18 peritoneal concentrations were found during acute bacterial peritonitis, in parallel with the increased infiltration of macrophages. Unexpectedly, CCL18 was also highly (50-fold) increased during sterile eosinophilic peritonitis, and peritoneal eosinophils were found to express CCL18.* In vitro* treatment of peritoneal macrophages with the vitamin D receptor agonist paricalcitol was able to reduce the secretion and the expression of CCL18 in isolated peritoneal macrophages. In conclusion, our study suggests that the chemokine CCL18 can be a mediator of peritoneal membrane failure associated with peritonitis episodes as well as providing a new potential therapeutic target.

## 1. Introduction

Peritoneal dialysis (PD) is a form of renal replacement used as an alternative to hemodialysis for the treatment of end-stage renal disease. It is based on the use of the peritoneum as a semipermeable membrane across which ultrafiltration and diffusion take place upon exposing it to bioincompatible, glucose-containing hyperosmotic solutions. However, continuous exposure to bioincompatible PD solutions causes acute and chronic inflammation and injury to the peritoneal membrane, which progressively leads to ultrafiltration failure (UFF) and irreversible structural fibrosis, compromising treatment efficacy and worsening patient outcomes [1, 2]. In some cases, PD-associated chronic peritoneal inflammation can result in encapsulating peritoneal sclerosis (EPS) [3], a fatal form of peritoneal inflammation characterized by a fibrous thickening of the peritoneum. Treatment for more than 4 years with bioincompatible PD solutions is known to present the highest risk for EPS [4, 5]. Additional risk factors for peritoneal membrane fibrosis are high glucose content and recurrent peritonitis episodes, which are a frequent complication in PD [4, 6–8]. Although bioincompatible solutions can themselves induce sterile peritoneal inflammation [9, 10] PD is affected by episodes of bacterial infection, leading to localized inflammation evidenced as peritonitis, a potentially destructive disease that exacerbates local peritoneal inflammation. Both acute and chronic peritoneal inflammation can lead to UFF [11]. Peritoneal inflammation is characterized by local upregulation of several cytokines and collagen synthesis by mesothelial cells and fibroblasts, leading to the loss of membrane integrity and fibrosis.

The successful repair of injured tissue requires a tightly controlled response to limit the structural alteration. Understanding the role of the various players involved can help in designing strategies aimed at limiting inflammation-mediated tissue injury.

The pathophysiological mechanisms that are involved in peritoneal functional impairment and UFF are not completely understood, although local conversion of mesothelial cells (MCs) by epithelial-to-mesenchymal transition (EMT) during the inflammatory and repair responses that are induced by PD might play a key role [12]. MCs and resident or infiltrating immune cells, including macrophages, monocytes, lymphocytes, and neutrophils, can produce a large number of cytokines, growth factors, and chemokines leading to membrane deterioration and fibrosis [10, 13].

Among leukocytes, alternatively activated macrophages (M2) are important players in tissue repair and the development of fibrosis [14] and might contribute to the fibrotic process of the peritoneum under PD [15].

The human chemokine CCL18, which has been involved in various progressive fibrotic disorders [16–19], is notably present at easily measurable levels in the effluent of patients treated with PD [15, 20]. CCL18 is also a hallmark of M2 macrophages in humans [14, 21]. High peritoneal CCL18 effluent levels in PD patients in relation to UFF and to predicting the future development of peritoneal sclerosis have recently been reported [15, 20]. Moreover, CD163+ M2 expressing CCL18 infiltrate the peritoneum in PD patients; their number increases during peritonitis episodes; and peritoneal macrophages isolated from patients with active infectious peritonitis synthesize high amounts of CCL18 when cultured* ex vivo* [15]. In a recent study, we reported that PD patients with sustained low levels of peritoneal CCL18 are at lower risk of developing peritoneal membrane dysfunction [22].

Given that CCL18 is involved in fibrotic processes and that recurrent peritonitis is a major risk factor for developing peritoneal membrane failure and peritoneal fibrosis, we sought to evaluate CCL18 protein concentrations in peritoneal effluents from patients experiencing peritonitis episodes. Pharmacological interventions aimed at diminishing the production of CCL18 were also explored. Among drug candidates, paricalcitol, a vitamin D receptor (VDR) agonist, has been shown to ameliorate fibrosis in a murine model of PD [23]. In humans, a preliminary study suggests that paricalcitol could increase the ultrafiltration capacity with a decrease of peritoneal protein losses in PD [24]. The absence of CCL18 in rodents precludes the study of paricalcitol effects on CCL18 peritoneal levels in the murine model. We herein analyzed the effect of this drug on CCL18 production by peritoneal macrophages.

CCL18 peritoneal concentrations were found to increase during the course of infectious bacterial peritonitis in parallel with the increased infiltration of macrophages. Unexpectedly, CCL18 effluent levels were also highly increased during the course of sterile eosinophilic peritonitis, and peritoneal eosinophils were found to express CCL18.

In addition,* in vitro* treatment of peritoneal macrophages with the VDR agonist paricalcitol was able to reduce the secretion and the expression of CCL18 in isolated peritoneal macrophages (pMΦs).

In conclusion, our study suggests that the chemokine CCL18 can be a mediator of peritoneal membrane failure associated with peritonitis episodes, as well as providing a new potential therapeutic target.

## 2. Patients and Methods

### 2.1. Patients

Patients were recruited from the Peritoneal Dialysis Unit of La Paz University Hospital in Madrid. The diagnosis of PD-associated peritonitis was based on the presence of abdominal pain, cloudy PD effluent with a leukocyte count above 100 cells/*μ*L, and a positive microbiological culture. The diagnosis of eosinophilic peritonitis (EP) was based on the presence of cloudy effluent (white blood cell count above 100 cells/*μ*L) with no infectious agent and eosinophils above 10%.

The study was approved by the Research Ethics Committee of La Paz University Hospital (PI12_0024). All clinical investigations were conducted according to the principles expressed in the declaration of Helsinki, and written informed consent was obtained from all the patients.

### 2.2. Cytokine Determinations

CCL18 was quantified using a DuoSet ELISA Development System (R&D Systems Europe, UK). IL-6 concentrations were measured by flow cytometry using the corresponding Cytometric Bead Array Flex Set kit (BD Biosciences).

### 2.3. Cytospin and Immunostaining

For cytospin preparations, peritoneal effluent cells were applied to slides at 10^5^ cells/slide. The cells were then fixed in 4% paraformaldehyde at room temperature. Sequential blocking was then performed with 0.03% hydrogen peroxide in methanol and in 10% normal goat serum/1% bovine serum albumin/0.1% saponin in Tris-buffered saline. Biotinylated goat anti-human CCL18 (R&D Systems) was added and incubated overnight at 4°C. Nonimmune serum was used as a negative background control staining. The sections were washed three times with phosphate-buffered saline and the signal was developed with peroxidase-conjugated streptavidin (R&D) and 3-3′-diaminobenzidine. Slides were counterstained with hematoxylin prior to visualization.

### 2.4. Cell Isolation and Treatment

Cells were isolated from PD effluents by centrifugation at 500*g* for 15 minutes at 4°C. Peripheral blood mononuclear cells (PBMCs) from control donors were prepared by Ficoll-Hypaque (Amersham Pharmacia Biotech, Sweden) density gradient centrifugation. pMΦs were obtained from PD effluent concentrates. Positive selection was performed by magnetic isolation using CD14 microbeads. For isolation of polymorphonuclear leukocytes FITC-conjugated CD66b-specific antibodies followed by anti-FITC microbeads (Miltenyi Biotec GmbH, Germany) were used as described previously [15]. Purity of macrophage fractions was at least 95%, as assessed by flow cytometry. CD14-APC, CD66b-FITC, and CD16-FITC antibodies (Miltenyi Biotec) were used for cytometry analysis of peritoneal cells.

Monocytes were isolated from PBMCs by the cell culture adherence method. For monocyte isolation by adherence, PBMCs were seeded onto cell culture plates and allowed to adhere in a 5% CO_2_ incubator at 37°C for 1 hour in serum-free medium. Nonadherent cells were removed, and the adherent cells were carefully washed twice with medium before culturing in RPMI medium plus 10% heat-inactivated fetal calf serum (FCS). Peripheral blood monocyte-derived M2 cells were generated as described [25]. Briefly, monocytes were incubated in RPMI with 10% heat-inactivated FCS and 50 U/mL IL-4 (Peprotech, UK) for 72 hours.

Cells were seeded at 200,000 cells/well in 96-well plates and incubated with various doses of paricalcitol. In some experiments, the commercial form (Zemplar®) was used as the source of drug, and cultures in the presence of the equivalent concentration of vehicle (30 : 50 : 20 propylene glycol : water : ethanol) were established in parallel as negative controls. Stock solutions of pure paricalcitol (Abbott) and vitamin D3 [1,25(OH)_2_D_3_] (Roche) were prepared in EtOH at 10^−3 ^M.

Cell viability was analyzed by exclusion of propidium iodide staining and flow cytometry.

### 2.5. Quantitative Reverse Transcription-Polymerase Chain Reaction (qRT-PCR)

Total RNA was isolated using the High Pure RNA Isolation Kit (Roche, Germany), and 1 *μ*g was reverse transcribed with random hexanucleotides and avian myeloblastosis virus (AMV) reverse transcriptase (Promega) in a final volume of 20 *μ*L for 1 hour at 42°C. For quantitative analysis of mRNA content, 1 *μ*L aliquots of the resulting cDNAs were PCR amplified in a Light Cycler (Roche) with FastStart DNA Master SYBR Green I (Roche) in duplicate. Primers used for PCR amplification were previously described [15]: CCL18 sense: 5′-ACA AAG AGC TCT GCT GCC TC-3′; CCL18 antisense: 5′-CCC ACT TCT TAT GGG GTC A-3′; B2M sense: 5′-CCA GCA GAG AAT GGA AAG TC-3′; B2M antisense: 5′-GAT GCT GCT TAC ATG TCT CG-3′. Standard curves for target mRNA expression were generated by amplifying 10-fold serial dilutions of known quantities of the specific PCR products. Quantification of target gene expression was obtained using Light Cycler system software. Relative units (RU) estimated from the quantification represent the ratio between CCL18 mRNA molecules and B2M mRNA molecules in each sample.

### 2.6. Statistical Analysis

The statistical analysis was performed using GraphPad Prism software (San Diego, CA). Parametric or nonparametric tests were used according to the distribution of the data. Data are shown as mean ± SEM, mean ± SD values, or median and interquartile ranges (IQR) as indicated in each figure. Data were analyzed with parametric or nonparametric *t*-tests for unpaired or paired samples. Correlation was assessed using Spearman correlation tests with *p* values. *p* values less than or equal to .05 were considered statistically significant.

## 3. Results

### 3.1. CCL18 Measurements in Peritonitis Effluent Samples

**Figure 1:**
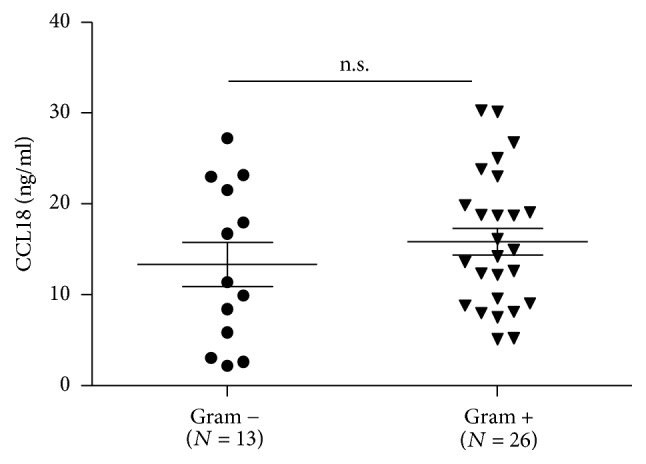
The concentration of CCL18 was evaluated in 39 peritoneal effluents from patients diagnosed with infectious peritonitis. Effluents were collected on the first day of cloudy effluent. Horizontal bars represent mean ± SEM values. No significant differences were found between Gram− (*N* = 13) and Gram+ bacteria (*N* = 26) infected cases (unpaired *t*-test).

Previous studies propose CCL18 as a marker and mediator of peritoneal fibrosis and UFF in patients treated with PD [15, 16]. Given recurrent peritonitis is a risk factor for peritoneal fibrosis and UFF, the concentration of CCL18 was evaluated in a group of 39 PD patients with infectious peritonitis. Effluent samples were obtained at diagnosis and the concentration of CCL18 was measured by enzyme-linked immunosorbent assay (ELISA). The mean concentration of CCL18 in peritonitis samples was 15.02 ± 7.87 ng/ml, whereas mean values previously reported ranged between 1 and 6 ng/ml. No significant differences were found in patients infected with Gram+ or Gram− bacteria. Mean observed values were 13.3 ± 2.43 ng/ml in the Gram− versus 15.86 ± 1.45 ng/ml in the Gram+ samples (Figure 1).

**Figure 2:**
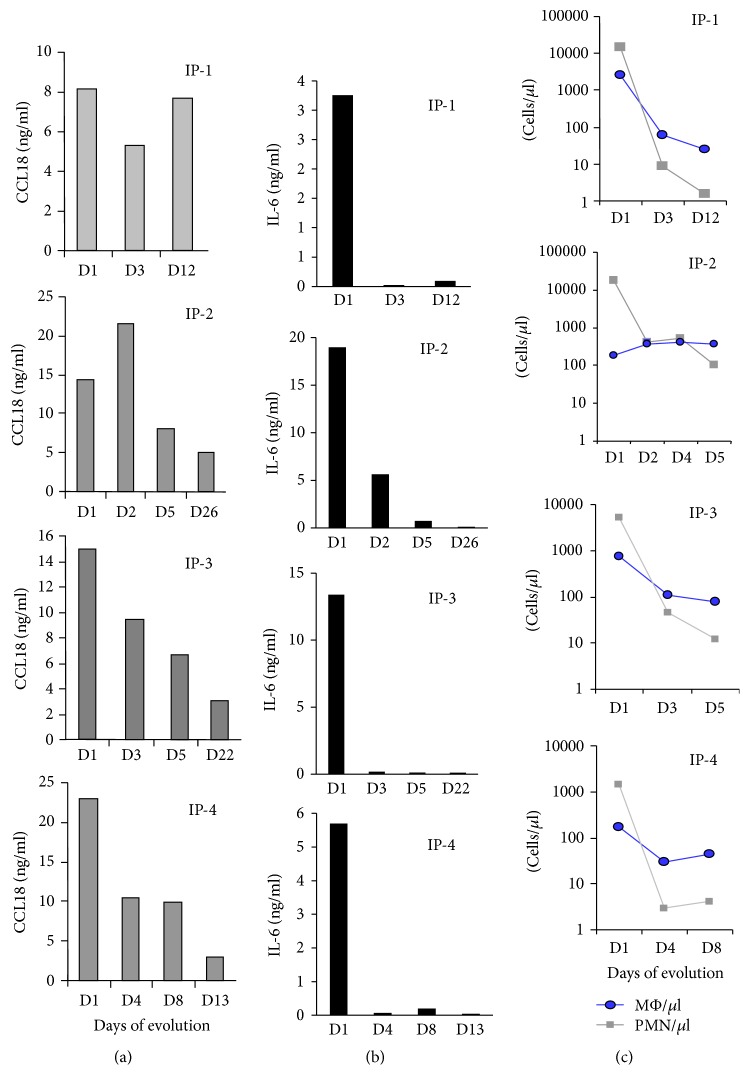
Serial cytokine concentrations and cell counts in peritoneal effluent during peritonitis episodes. (a) Evolution of CCL18 effluent levels in serial samples from four patients with infectious peritonitis (IP) shown as IP-1 to IP-4. (b) Evolution of IL-6 effluent levels in serial samples from the same patients. (c) Comparison of neutrophils (PMN) and macrophage (MΦ) cell counts in serial effluent samples. Differential cell counts on D26 (IP-2), D22 (IP-3), and D13 (IP-4) were not available given that the total cell counts were <50 cells/*μ*l in those samples.

We evaluated CCL18 concentrations in serial samples from four of the PD patients with infectious peritonitis (IP) (Figure 2(a)). CCL18 effluent concentrations showed the highest values during the first days of peritonitis and decreased slowly following a kinetics similar to absolute macrophage counts in these samples (Figure 2(c)) and in agreement with previous reports describing macrophages as the primary source of this chemokine [15].

**Figure 3:**
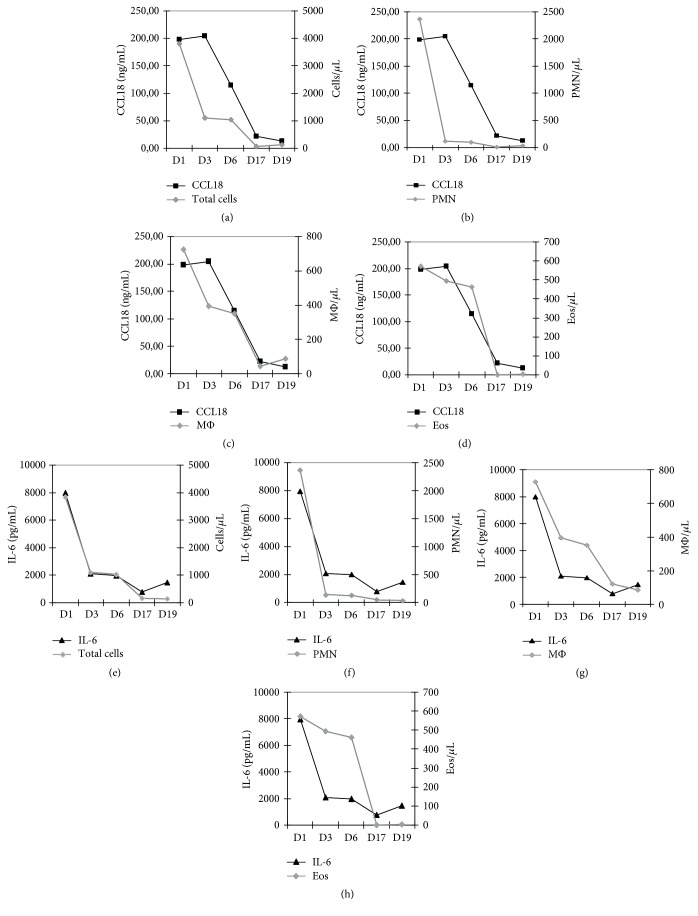
Kinetics of effluent cell counts and cytokine concentrations during EP. (a–d) CCL18 effluent levels and cell counts in serial effluent samples. (e–h) IL-6 effluent levels and cell counts in serial effluent samples.

Serial samples were also analyzed in one additional case of sterile EP associated with an accidental exposure of the peritoneum to chlorhexidine. Surprisingly, whereas the mean CCL18 concentrations on day 1 and on days 2–4 in the IP samples were 15.5 ± 6.1 ng/ml and 11.7 ± 6.9 ng/ml, respectively, in the EP samples, the concentration of CCL18 was 198.12 ng/ml on day 1 and 204.65 ng/ml on day 3 (Figure 3 and Supplemental Figure 1(A)). IL-6 was also evaluated for comparative purposes as the hallmark cytokine in inflammatory processes and in PD-associated peritonitis [26]. Elevated levels of IL-6 were found in spent effluents from all patients on the first day of peritonitis, with similar values in IP samples and in the EP sample (Supplemental Figure 1(B)). A sharp decline in IL-6 occurred in all the IP cases, as previously described [26, 27] (Figure 2(b)), and in contrast to CCL18 levels, which remained elevated during the following days and declined more slowly (Figure 2(a)).

### 3.2. Peritoneal Eosinophils Are a Source of CCL18 in Eosinophilic Peritonitis

Cytokine concentrations were compared with absolute and differential cell counts in effluent samples from the patient with EP (Figure 3). A significant correlation was found between the kinetics of CCL18 concentration and the macrophage counts in the EP samples (*r* = 0.889; *p* = .043) (Figure 3(c)). However, the strongest correlation was found between CCL18 effluent levels and eosinophils (*r* = 0.949; *p* = .013), with peak CCL18 concentrations detected in effluents with the highest absolute eosinophil count (Figure 3(d)). In contrast, IL-6 effluent levels paralleled polymorphonuclear neutrophils (PMNs) and absolute cell numbers in effluent samples from EP (Figures 3(e) and 3(f)), and no correlation was observed between effluent IL-6 concentrations and eosinophil cell count (Figure 3(h)).

**Figure 4:**
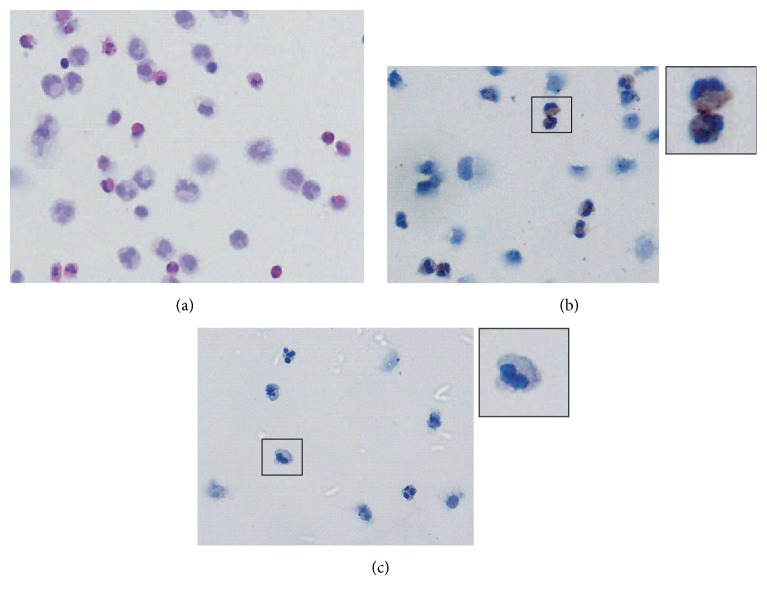
Cytospin preparations showing effluent cell content in EP. (a) Hematoxylin and eosin staining. (b) Immunocytochemistry showing CCL18 staining of eosinophils. (c) Background staining with nonimmune serum.

Although CCL18 is thought to be primarily produced by M2 macrophages involved in tissue repair and fibrosis and by dendritic cells [28], eosinophils have occasionally been reported to produce CCL18 during inflammatory conditions [29, 30]. The kinetics of CCL18 effluent concentrations in EP and eosinophil counts suggested that not only macrophages but also eosinophils might contribute to the production of CCL18 in this case. To test this hypothesis, immunostaining of cytospin preparations from eosinophilic effluents was performed. Specific staining was detected in the cytoplasm of typical binucleated cells, supporting the role of eosinophils as a source of CCL18 in this case (Figure 4).

**Figure 5:**
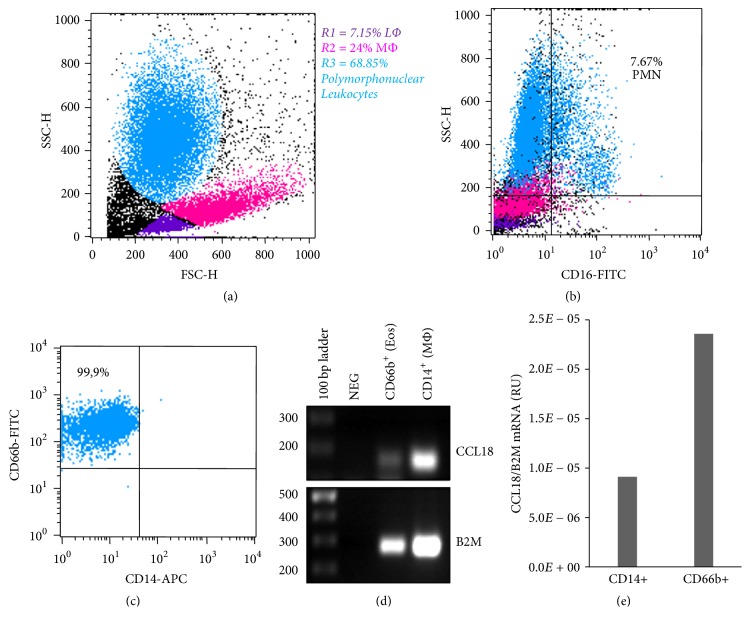
(a) Flow cytometry analysis of peritoneal effluent leukocytes on D6 according to morphological parameters. LΦ: lymphocytes; MΦ: macrophages. (b) Flow cytometry analysis of CD16 expression in peritoneal effluent cells on D6. The cutoff level for positive staining was set according to isotype control background staining. PMN: polymorphonuclear neutrophils. (c) Flow cytometry analysis of CD66b^+^ polymorphonuclear cells isolated from peritoneal effluent cells. (d) RT-PCR of CCL18 gene expression in CD66^+^ and CD14^+^ purified peritoneal leukocytes. (e) Quantitative RT-PCR analysis of CCL18 gene expression in CD66+ and CD14+ purified peritoneal leukocytes.

In a previous report we found that only peritoneal macrophages were able to produce CCL18 during infectious peritonitis, given that the cytokine was not detected in culture supernatants from neutrophils (PMNs) or lymphocytes [15]. To further analyze the putative role of eosinophils as producers of CCL18 in the patient with EP, CD66b^+^ polymorphonuclear leukocytes (among which 85% were eosinophils, as assessed by CD16 negative staining of eosinophils versus CD16 positive neutrophils) were isolated from peritoneal effluent cells on D6, as well as CD14^+^ macrophages for comparative purposes (Figures 5(a)–5(c)). CCL18 transcripts were detected in both the CD66b^+^ and CD14^+^ fractions (Figure 5(d)). Quantitative PCR analysis confirmed CCL18 mRNA transcripts in polymorphonuclear leukocytes in this case (Figure 5(e)). Altogether, the data strongly suggest that eosinophils are producers of CCL18 in EP.

### 3.3. Paricalcitol Downregulates the Production of CCL18 by Peritoneal Macrophages

**Figure 6:**
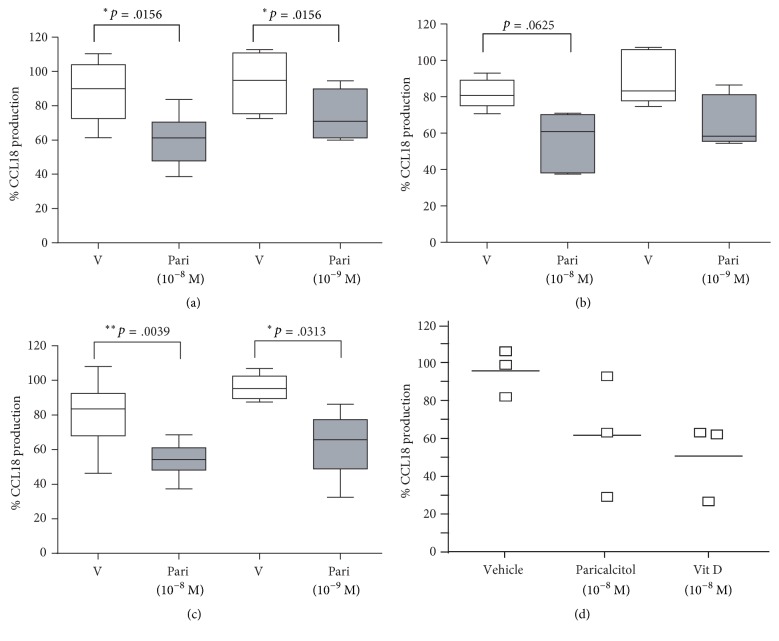
(a–c) Peritoneal macrophages isolated from patients experiencing peritonitis episodes were treated with vehicle or Zemplar in doses equivalent to 10^−8 ^M and 10^−9 ^M of paricalcitol during 24 h (a), 48 h (b), or 72 h (c). Equivalent cell cultures were established in the absence of any treatment. The concentration of CCL18 was evaluated in cell-free supernatants, and the production of CCL18 in the presence of vehicle or drug referred to the production in cell culture media alone. Differences between cultures in the presence of paricalcitol or vehicle were analyzed by the Wilcoxon matched pairs rank test. (d) Peritoneal macrophages were treated with paricalcitol as pure substance, vitamin D, or vehicle (ethanol) for 48 h.

Paricalcitol, a VDR agonist, has been shown to ameliorate peritoneal fibrosis in a murine model of PD [23]. Given that CCL18 has been involved in peritoneal fibrosis in PD patients [15, 20, 22] we tested the effect of paricalcitol in the production of CCL18 by human pMΦs isolated from PD effluents drawn from patients suffering from peritonitis and cultured with various doses of nontoxic concentrations of Zemplar as a source of paricalcitol or vehicle (supplementary Figure 2). As shown in Figures 6(a)–6(c), pharmacological treatment was able to significantly downregulate the production of CCL18 in these cells at concentrations equivalent to 10^−8 ^M and 10^−9 ^M of paricalcitol. This effect was obtained as early as 24 hours, and it was also observed at 48 hours and 72 hours of cell culture. Similar results were obtained with the pure substance and with equivalent doses of vitamin D3 (Figure 6(d)).

**Figure 7:**
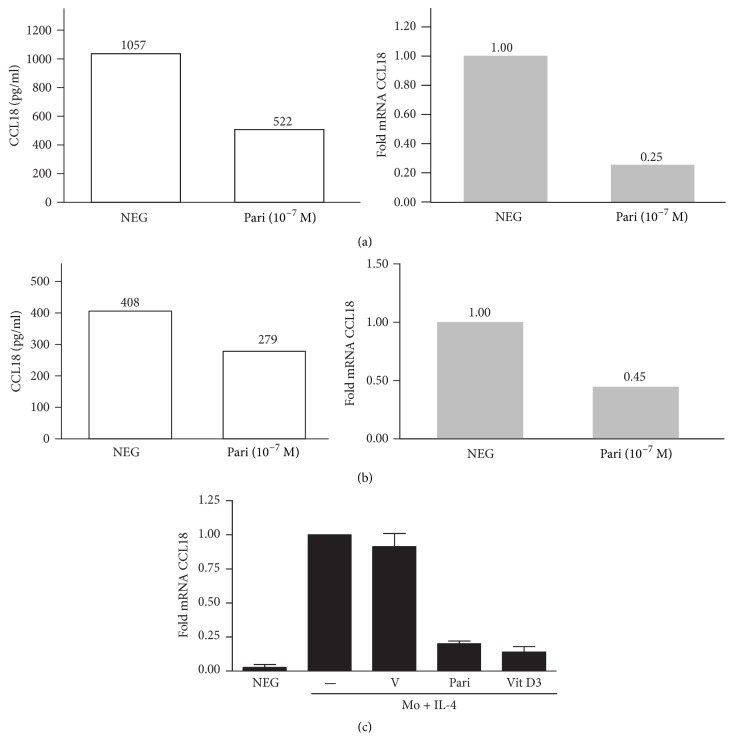
(a) Peritoneal macrophages from a patient with recent infectious peritonitis were treated with paricalcitol or vehicle for 24 h. The protein concentration of CCL18 was evaluated in cell culture supernatants by ELISA, and CCL18 mRNA levels were estimated by quantitative RT-PCR in the same cultures. (b) Peritoneal macrophages from an uninfected peritoneum were isolated from the peritoneal lavage upon catheter insertion and treated as in (a). (c) Peripheral blood monocytes were pretreated with vehicle (V), 10^−7 ^M paricalcitol (Pari), or vitamin D3 for 24 h. The cells were treated thereafter with IL-4 for 3 days before RNA extraction and evaluation expression of CCL18. mRNA levels by quantitative RT-PCR. Mean ± SD values from four independent experiments are shown.

It has been suggested that the regulation of CCL18 protein synthesis and secretion can be a complex process with some degree of posttranscriptional regulation [28]. To analyze whether VDR agonists regulate CCL18 mRNA levels, quantitative RT-PCR assays were performed in isolated pMΦs treated with paricalcitol. CCL18 mRNA levels were downregulated in parallel with the diminished production of CCL18 protein in pMΦs from two different donors representing infected and uninfected peritoneal samples (Figures 7(a) and 7(b)). Given that IL-4 is one of the main inducers of CCL18 [31], we tested if VDR agonists could modulate the expression of the chemokine in M2 macrophages generated* in vitro* upon treatment of peripheral blood monocytes with IL-4. As shown in Figure 7(c), the expression of CCL18 was highly upregulated in monocyte-derived macrophages treated for 3 days with IL-4, and pretreatment with paricalcitol or vitamin D was able to prevent the upregulation of CCL18 in these cells.

## 4. Discussion

Peritonitis episodes constitute a frequent complication in PD patients, and recurrent peritonitis is a well-known clinical risk factor for peritoneal membrane dysfunction and EPS.

There is an unmet requirement for biomarkers as tools to identify patients who are at higher risk of technique failure to guide interventions that could improve clinical outcomes [32]. Soluble factors released during inflammatory processes, such as IL-6 and IL-17, have been proposed as biomarkers and mediators of peritoneal membrane deterioration (reviewed in [32, 33]). However, none are used in the clinical routine, and some cytokines, such as IL-17, are not easily measured in PD effluents in humans. We propose CCL18 as a biomarker of peritoneal membrane outcome in PD [22].

Given that high levels of the human chemokine CCL18 have been shown to be related to peritoneal membrane failure and fibrosis [15, 20, 22], whereas sustained low CCL18 peritoneal concentrations appear to be protective [22], we evaluated CCL18 levels in the peritoneum of patients undergoing peritonitis episodes. We found that the peritoneal concentration of CCL18 was increased approximately 5-fold in effluents from patients with infectious peritonitis compared with reported values in uninfected samples. In a previous study involving a longitudinal analysis of 43 patients, CCL18 effluent concentrations ranged from 2.8 ± 1.6 ng/ml at the start of dialysis to 3.4 ± 1.8 ng/ml after 3 years of PD treatment [22]. In a cross-sectional analysis of 61 patients treated for more than 3 years with PD mean effluent, CCL18 values were 3.95 ± 2.6 ng/ml [22]. The high concentrations during IP are consistent with a previous finding showing that pMΦs from patients undergoing peritonitis or who recently had peritonitis were able to secrete higher amounts of CCL18 compared with pMΦs from uninfected patients [15].

Unexpectedly, in a patient identified with sterile eosinophilic peritonitis, the peritoneal effluent was extremely rich in CCL18, with levels approximately 10-fold higher than those found in patients with IP and 50-fold more than those found in uninfected samples, whereas, effluent levels of IL-6, which were evaluated in the same samples for comparative purposes, were similar on day 1 in both the IP and EP samples. The differences between a peritoneal inflammatory recognized marker, IL-6, and CCL18 during peritonitis episodes should be remarked. It is of note that the peritoneal concentrations of CCL18 remained elevated for several days, whereas there was a sharp decrease in IL-6. Both IL-6 and CCL18 have been reported to increase in different inflammatory conditions, some leading to tissue fibrosis [34–37]. As a main difference, although CCL18 is primarily produced by cells of myeloid origin, multiple cell types are producers of IL-6, with endothelial cells being an important source of this cytokine. Infiltrating neutrophils as well as mesothelial cells could also be an important source of IL-6 during bacterial peritonitis [37]. The analysis of serial samples in four patients with IP showed that the concentrations of CCL18 paralleled the numbers of macrophages infiltrating the peritoneum and are consistent with previous descriptions of macrophages and mononuclear phagocytes as the primary producers of CCL18 [21]. Cell counts in the serial samples from our case of EP suggest that the peritoneal eosinophilic infiltrate was likely contributing to the peritoneal production of CCL18. Evidences of CCL18 production by activated peritoneal eosinophils in this case are cytospin analysis of CCL18 cytoplasmic staining in eosinophils and gene expression data in polymorphonuclear leukocytes among which 85% were eosinophils.

Although macrophages are typically reported as the major source of this chemokine [21], CCL18 protein production by eosinophils has occasionally been described as being related to the activation status of these cells, given that CCL18 was found only in eosinophil culture supernatants from eosinophilic donors, but not from individuals with nonactivated eosinophils [29]. Our data support the activated status of eosinophils in EP, given that strong staining of CC18 was detected in peritoneal eosinophils. Eosinophils have also been related to tissue fibrosis in several diseases and are a source of several profibrotic mediators that alter fibroblast activity and tissue remodeling [38, 39]. Some of these mediators could also stimulate the profibrotic activity of pMΦ. The production of CCL18 by eosinophils in EP provides a new additional explanation for the association of recurrent EP during PD with UFF and potentially peritoneal fibrosis [40].

As part of its profibrotic activity, CCL18 has been shown to stimulate collagen deposition [41] and fibroblast proliferation [42]. In consonance with these actions, circulating CCL18 levels have been demonstrated to be a biomarker for disease activity and outcome in various fibrotic diseases [19, 43, 44], as well as in some eosinophilic disorders [30, 45, 46]. Moreover, CCL18 has been shown to promote EMT in various cell systems [47–50]. It is thus possible that it could also contribute to the EMT of mesothelial cells, which has been proposed as one of the mechanisms leading to peritoneal fibrosis and membrane failure [12].

In terms of peritoneal fibrosis, CCL18 has been demonstrated to herald the development of peritoneal functional deterioration in PD patients [15, 20], in whom the ability of peritoneal macrophages to stimulate fibroblast proliferation is correlated with CCL18 mRNA levels. Moreover, recombinant CCL18 was able to stimulate the proliferation of peritoneal fibroblasts [15].

Several molecules have been tested for their ability to prevent peritoneal fibrosis* in vitro* and in animal models (reviewed in [8]). Among them, paricalcitol is a selective VDR agonist. VDR is a nuclear hormone receptor expressed in various cell types, including those of the immune system. VDR agonists have been shown to modulate immune responses, inflammation, and fibrosis [51].

Considering the role played by inflammation in peritoneal fibrosis during PD, we studied the effects of paricalcitol in the production of CCL18. We found that the release and gene expression of CCL18 were downregulated in pMΦs and in* in vitro*-differentiated human M2 cells, which are the primary producers of CCL18. These results appear to contrast with previous data showing upregulation of CCL18 by vitamin D in monocyte-derived immature dendritic cells [52]. However, recent reports demonstrate that the modulation of immune signaling by vitamin D in human mononuclear phagocytes is cell-type-specific, with differential responses in monocytes, macrophages, and dendritic cells [53]. It is thus possible that opposite effects are found on the regulation of CCL18 by VDR agonists in different cellular systems.

On the other hand, in a mouse model of PD, intraperitoneal administration of paricalcitol was shown to reduce peritoneal fibrosis and ultrafiltration failure [23]. In that model, the beneficial effects appeared to be dependent on a reduction of intraperitoneal concentrations of IL-17. The lack of rodent orthologues of human CCL18 [28] precludes the investigation of CCL18 and its involvement in fibrosis in murine models. On the other hand, IL-17 is highly diluted in human peritoneal effluents. The need to concentrate the samples for measurement of this cytokine hampers accurate determinations in human samples.

A preliminary study involving 23 patients on PD suggests that paricalcitol could improve the ultrafiltration capacity with less peritoneal protein loss [24]. The authors suggest an effect of VDR upon the renin-angiotensin-aldosterone system, but it is tempting to speculate that lower peritoneal concentrations of CCL18 might be present in paricalcitol-treated patients. In this sense, a slight reduction in the peritoneal concentration of CCL18 was observed in six patients who were more than 3 years on PD and treated with paricalcitol compared with a set of 31 patients of the same vintage (data not shown).

## 5. Conclusion

In conclusion, our study suggests that the chemokine CCL18 can be a mediator of peritoneal membrane failure associated with peritonitis episodes and might provide a new potential therapeutic target.

Further clinical studies are needed to confirm the usefulness of VDR agonists to preserve the peritoneal membrane and to analyze the putative mechanisms involved.

## Supplementary Materials

Supplementary Materials Supplemental Figure 1: serial cytokine concentrations and cell counts in peritoneal effluent during infectious peritonitis and eosinophilic peritonitis. (A) Comparison of CCL18 concentration in serial effluent samples from infectious peritonitis (IP) and eosinophilic peritonitis (EP). (B) IL-6 effluent levels in serial samples from IP and EP. D1–D26 indicate days of evolution. Supplemental Figure 2: dose-dependent effects of VDR agonists on cell viability. Cell viability of human monocytes was assessed by exclusion of propidium iodide staining and analyzed by flow cytometry. Results are shown as mean ± SD values of triplicates. (A)–(C) Viability of monocytes upon culture during 24, 48, or 72 hours with decreasing concentrations of Zemplar or equivalent vehicle (30:50:20 propylene glycol:water:ethanol). (D)–(F) Viability of monocytes upon culture during 24, 48, or 72 hours with different concentrations of paricalcitol (pure substance), vitamin D, or equivalent vehicle (EtOH).
