# Supplementary material for: Prominent Levels of the Profibrotic Chemokine CCL18 during Peritonitis: In Vitro Downregulation by Vitamin D Receptor Agonists
Source: Biomed Res Int. 2018 Apr 4;2018:6415892. doi: 10.1155/2018/6415892 (PMC5904802; doi:10.1155/2018/6415892)
Supplement: Supplementary Materials — Supplemental Figure 1: serial cytokine concentrations and cell counts in peritoneal effluent during infectious peritonitis and eosinophilic peritonitis. (A) Comparison of CCL18 concentration in serial effluent samples from infectious peritonitis (IP) and eosinophilic peritonitis (EP). (B) IL-6 effluent levels in serial samples from IP and EP. D1–D26 indicate days of evolution. Supplemental Figure 2: dose-dependent effects of VDR agonists on cell viability. Cell viability of human monocytes was assessed by exclusion of propidium iodide staining and analyzed by flow cytometry. Results are shown as mean ± SD values of triplicates. (A)–(C) Viability of monocytes upon culture during 24, 48, or 72 hours with decreasing concentrations of Zemplar or equivalent vehicle (30:50:20 propylene glycol:water:ethanol). (D)–(F) Viability of monocytes upon culture during 24, 48, or 72 hours with different concentrations of paricalcitol (pure substance), vitamin D, or equivalent vehicle (EtOH). [file 6415892.f1.pdf]

Supplemental Figures

A

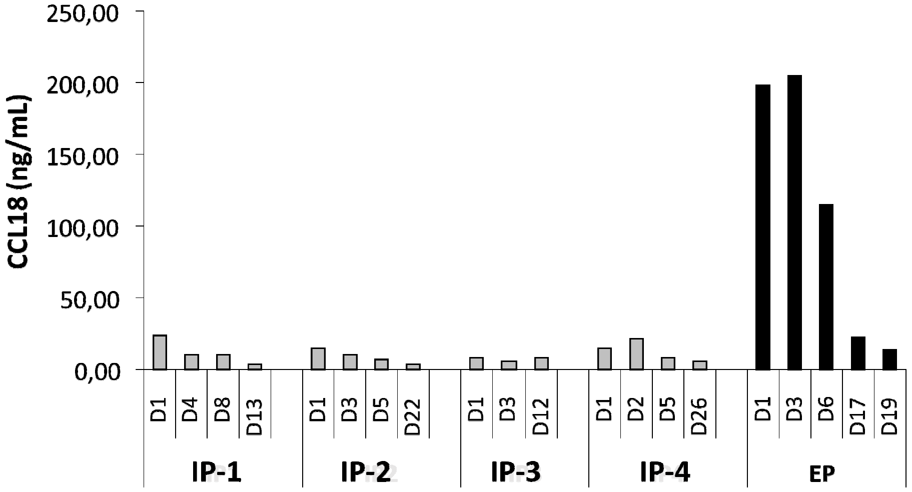

B

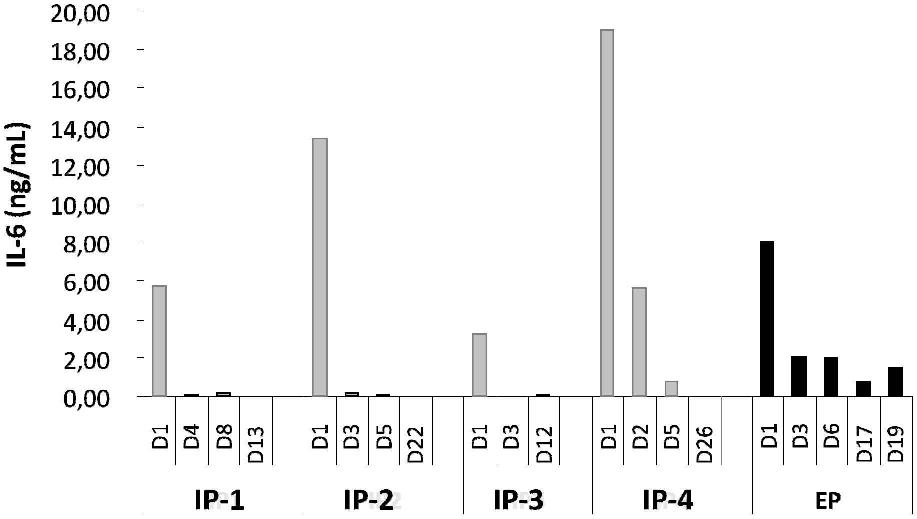

Supplemental Figure 1. Serial cytokine concentrations and cell counts in peritoneal effluent during infectious peritonitis and eosinophilic peritonitis. (A) Comparison of CCL18 concentration in serial effluent samples from infectious peritonitis (IP) and eosinophilic peritonitis (EP). (B) IL-6 effluent levels in serial samples from IP and EP. D1–D26 indicate days of evolution.

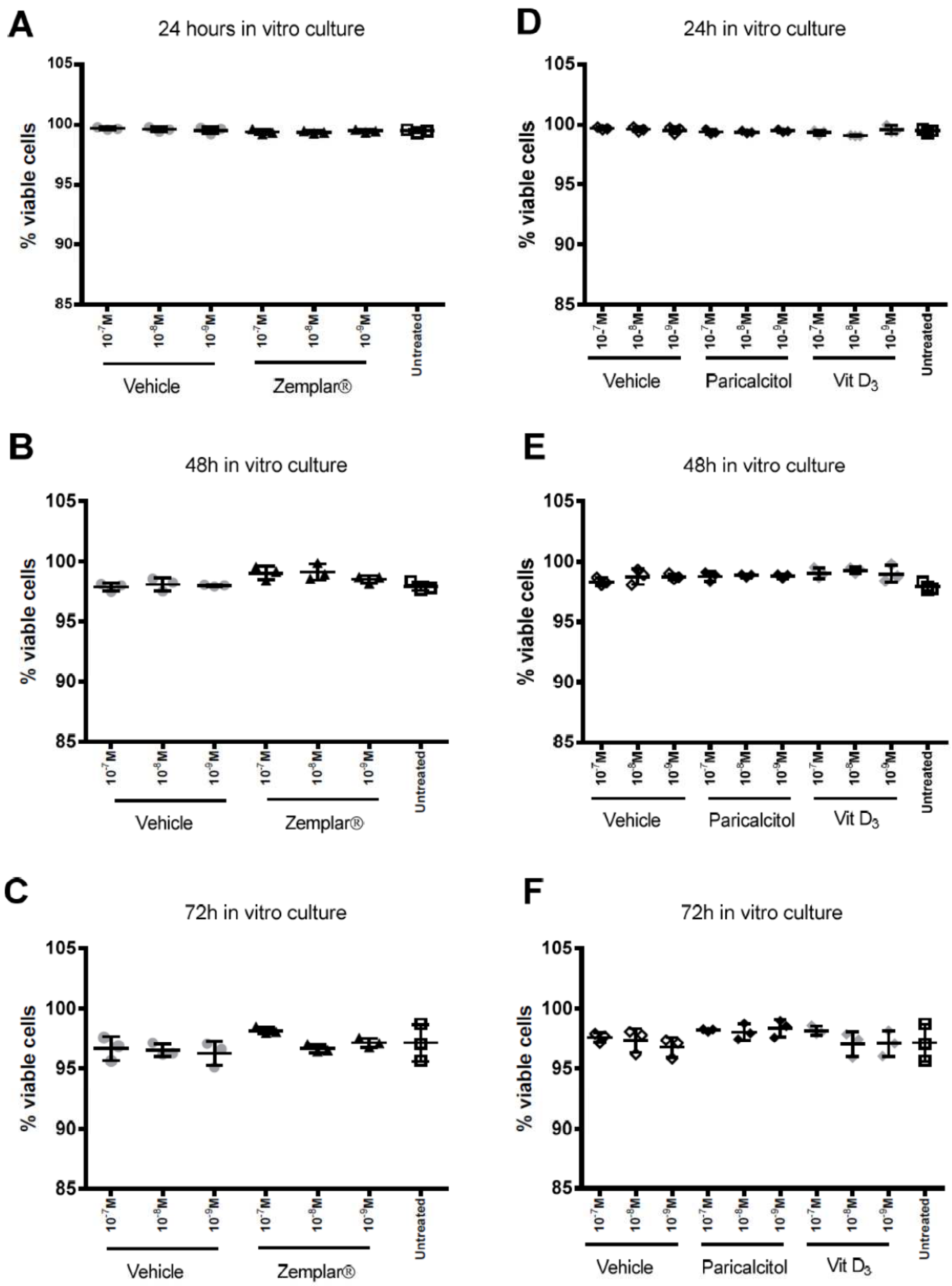

Supplemental Figure 2. Dose-dependent effects of VDR agonists on cell viability. Cell viability of human monocytes was assessed by exclusion of propidium iodide staining and analyzed by flow cytometry. Results are shown as mean ±SD values of triplicates. A-C: viability of monocytes upon culture during 24, 48 or 72 hours with decreasing concentrations of Zemplar® or equivalent vehicle (30:50:20 propylene glycol:water:ethanol). D-F: viability of monocytes upon culture during 24, 48 or 72 hours with different concentrations of paricalcitol (pure substance), vitamin D, or equivalent vehicle (EtOH).
